# Supplementary material for: HER2-Targeted Immunotherapy and Combined Protocols Showed Promising Antiproliferative Effects in Feline Mammary Carcinoma Cell-Based Models
Source: Cancers (Basel). 2021 Apr 21;13(9):2007. doi: 10.3390/cancers13092007 (PMC8122524; doi:10.3390/cancers13092007)
Supplement: Supplementary file 1 [file cancers-13-02007-s001.zip › cancers-1151266-supplementary.pdf]

cancers

MDPI

Supplementary Materials: HER2-Targeted Immunotherapy and Combined Protocols Showed Promising Antiproliferative Effects in Feline Mammary Carcinoma Cell-Based Models

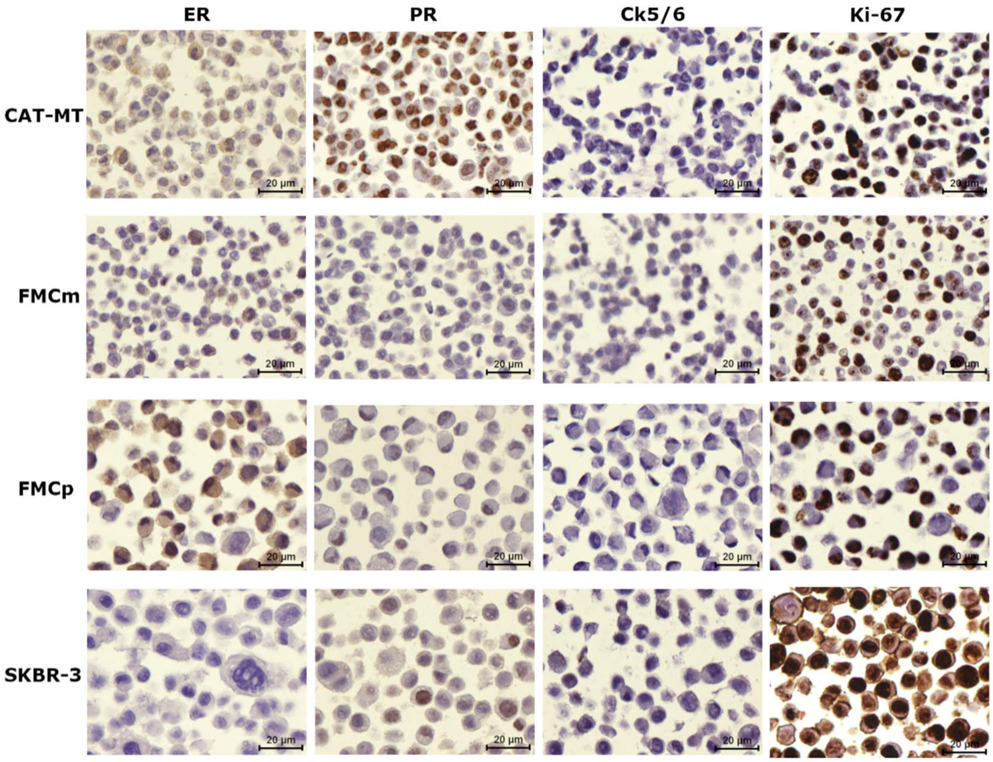

Figure S1. Characterization of the FMC cell lines (CAT-MT, FMCm and FMCp) and the human SKBR-3 cell line, by immunocytochemistry.

Table S1. Summary of the characterization of the FMC (CAT-MT, FMCm and FMCp) and human breast cancer (SKBR-3) cell lines, and its origin.

<table>
<tr><th>Cell Line</th><th>Tumor Origin</th><th>ER *</th><th>PR **</th><th>Ck5/6</th><th>Ki-67 (%)</th></tr>
<tr><td>CAT-MT</td><td>Mammary adenocarcinoma</td><td>3</td><td>8</td><td rowspan="4">Negative</td><td>50.2</td></tr>
<tr><td>FMCm</td><td>Metastatic lymph node</td><td>3</td><td>0</td><td>68.5</td></tr>
<tr><td>FMCp</td><td>Primary breast tumor</td><td>7</td><td>0</td><td>57.4</td></tr>
<tr><td>SKBR-3</td><td>Mammary adenocarcinoma</td><td>0</td><td>4</td><td>53.6</td></tr>
</table>

* ER—estrogen receptor; ** PR—progesterone receptor.

Table S2. Analysis of *her2* ECD mutations, subdomains II and IV, which encode for the regions recognized by pertuzumab and trastuzumab, respectively, in the feline mammary carcinoma clinical samples.

Region	Exon	Nucleotide Position (bp)	Mutations Frequency	Breed[1] (n)	Age Class [2] (n)	Size Class [3] (n)	Tumor Sub-type (n)	Stage (n)	mRNA (bp); aa Modification	Observations *
II	3	7554	2	Ind (2)	2; 3	2 (2)	LB (2)	2; 3	231; Ile	Synonymous; apolar
		7563	2	Ind (2)	2 (2)	1; 2	LB; LB-HER2	1; 2	240; Val	Synonymous; apolar
		7568	4	Ind (3)	2 (2); 3	1 (2); 2 (2)	LB (3); LB-HER2	1 (2); 2; 3	245; Gly to Val	Apolar
		7569	1	Ind	2	2	LB-HER2	3	246; Gly	Synonymous; apolar
		7572	2	Ind; SM	2 (2)	2; 3	LB (2)	2; 3	249; Tyr	Synonymous; polar neutral
		7579	1	Ind	2	2	LB	2	256; Ile to Leu	Apolar
		7587	1	Ind	3	2	LB	3	246; His to Gln	Polar basic to polar neutral
		7595	1	Ind	2	2	LB	2	272; Val to Glu	Apolar to polar acid
		7609	1	Ind	2	2	LB-HER2	3	286; Leu	Synonymous; apolar
		7618	5	Ind (5)	2 (4); 3	1; 2 (2); 3 (2)	LB (4); LB-HER2	1; 3 (3); 4	295; Leu to Val	Apolar
		7626	1	Ind	3	2	LB	3	303; Ile to Met	Apolar
		7644	4	Ind (4)	2 (4)	2 (2); 3 (2)	LB (3); LB-HER2	2 (2); 3; 4	321; Leu	Synonymous; apolar
		7656	6	Ind (4); SM; NF	2 (6)	1; 2 (2); 3 (3)	LB (5); LB-HER2	1; 2 (2); 3 (2); 4	333; Asn to Lys	Polar neutral to polar basic
		7668	1	Ind	2	2	LB	3	345; Ala	Synonymous; apolar
		7672	3	Ind (2); NF	2 (3)	1; 2; 3	LB (3)	1; 2; 3	349; Leu	Synonymous; apolar
		7674	1	Ind	2	2	LB-HER2	3	351; Leu	Synonymous; apolar
		7690	1	Ind	2	2	LB	2	367; Leu/Leu to Val	Heterozygous; synonymous/or change of codon; apolar
		7696	1	Ind	3	2	LB	3	373; Asn to Thr	Polar neutral
		7706	1	Ind	2	1	LB	1	383; Pro to His	Apolar to polar basic
		7711	1	Ind	2	2	LB	3	388; Thr to Ala	Polar neutral to apolar
		7718	2	Ind (2)	2 (2)	1; 2	LB; LB-HER2	1; 2	395; Ala to Gly	Apolar
		7727	1	Ind	2	2	LB	3	404; Gly to Ala	Apolar
		7735	1	NF	2	3	LB	3	412; Arg	Synonymous; polar basic
		7741	4	Ind (3); SM	2 (4)	1; 2 (2); 3	LB (2); LB-HER2 (2)	1; 2; 3 (2)	418; Leu to Val	Apolar
		7756	3	Ind (2); NF	2 (3)	1; 2; 3	LB (3)	1; 3; 4	433; Leu to Phe	Heterozygous; apolar
	4	No mutations reported in the sequenced regions, from c.8575bp to c.8709bp								

	**10**	13320	2	Ind; Per	2 (2)	2 (2)	LB; TN	4 (2)	1193; Arg to Ile	Heterozygous; polar basic to apolar
	**11**	13435	1	Ind	2	2	TN	4	1233; Tyr	Heterozygous, synonymous; polar neutral
		13451	2	Ind (2)	2 (2)	2 (2)	LB; TN	3; 4	1249; His to Asn	Heterozygous; polar basic to polar neutral
		13458	1	Ind	2	2	TN	4	1256; Leu to Ser	Heterozygous; apolar to polar neutral
**IV**		13464	1	Ind	2	2	TN	4	1262; Leu to Ser	Apolar to polar neutral
		13485	1	Ind	2	2	TN	4	1283; Leu to His	Apolar to polar basic
	**12**	13772	1	Ind	2	3	LB-HER2	3	1365; Leu	Synonymous; apolar
		13917	1	Ind	1	1	LB-HER2	1	1510; Cys to Gly	Polar neutral to apolar
	**13**	14299	1	Ind	2	1	LB	1	1536; Tyr	Synonymous; polar neutral
		14403	1	Ind	3	1	TN	1	1640; Leu to Arg	Apolar to polar basic
		14406	1	Ind	2	1	LB	1	1643; Gln to Pro	Base insertion; polar neutral to apolar

[1] Ind—indeterminate; SM—Siamese; NF—Norwegian Forest Cat; Per—Persian. [2] Age class 1< 8 years; 2–8 to 12 years; 3> 12 years. [3] Size class 1< 2cm; 2—2 to 3cm; 3> 3 cm. *Mutations not defined were homozygous.

Table S3. Feline cell lines presented a small number of mutations in the feline *her2* ECD, subdomains II (exons 3 and 4) and IV (exons 10 to 13), with none of them reported in human as leading to resistance to therapy. DNA samples were acquired from cells in culture, and PCR and sequencing were performed with specific primers.

Subdomain	Exon	Cell Line	gDNA Mutation (bp)	Protein Change
**II**	**3**	CAT-MT	c.7684 G > A	Asp to Asn<br>Polar acid to polar neutral
		FMCm	c.7622 G > A	Arg to Gln<br>Polar basic to apolar
		FMCp	c.7554 C > A	Ile<br>Apolar (synonymous)
			c.7568 G > T	Gly to Val<br>Apolar
			c.7572/c.7573 TG > CC	Tyr/Val to Leu<br>Polar neutral to apolar
			c.7644 C > A	Leu<br>Apolar (synonymous)
			c.7647 T > A	Phe to Leu<br>Apolar
			c.7656 C > G	Asn to Lys<br>Polar neutral to polar basic
			c.7668 C > A	Ala<br>Apolar (synonymous)
			c.7672 C > T	Leu<br>Apolar (synonymous)
			c.7756 C > T	Leu to Phe<br>Apolar
	**4**	No mutations reported		
**IV**	**10**	FMCp	c.13303 G > A	Ala to Thr
				Apolar to polar neutral
			c.13345 T > C	Ile<br>Apolar (synonymous)
	**11**	FMCp	c.13470 T > C	Leu to Ser<br>Apolar to polar neutral
			c.13514 C > A	Ala<br>Apolar (synonymous)
	**12**	FMCm	c.13836 G > A	Val to Ile<br>Apolar (heterozygous)
	**13**	No mutations reported		
